# Longitudinal evaluation of advanced glaucoma: ten year follow-up cohort study

**DOI:** 10.1038/s41598-023-50512-7

**Published:** 2024-01-04

**Authors:** Young In Shin, Yoon Jeong, Min Gu Huh, Young Kook Kim, Ki Ho Park, Jin Wook Jeoung

**Affiliations:** 1 Department of Ophthalmology, Seoul National University College of Medicine, Seoul, Korea; 2 Department of Ophthalmology, Seoul National University Hospital, 101 Daehak-ro, Jongno-gu, Seoul, 03080 Korea

**Keywords:** Eye diseases, Risk factors

## Abstract

This study focused on patients with advanced open-angle glaucoma (OAG) and aimed to identify key factors for monitoring them. We included 127 such patients who were followed for seven years or more, undergoing annual ophthalmic examinations. Glaucoma progression was defined as a deterioration in either structure or function. The progression rates and risk factors were evaluated. The patients were divided into upper- and lower-half subgroups based on the reduction in intraocular pressure (IOP) from the baseline. Over an 11-year period, glaucoma progression was detected in 59 eyes (46.5%). The rate of change in mean deviation (MD) was − 0.43 dB/year for the entire population; − 0.67 dB/year for progressors; and − 0.20 dB/year for non-progressors. Hypertension and disc hemorrhage (DH) were more common in progressors compared to non-progressors (45.8 vs. 23.5%, 11.9 vs. 1.5%; *P* = 0.008 and *P* = 0.016). Multivariate Cox’s proportional hazard model revealed that the presence of DH and a better baseline MD were associated with glaucoma progression. Additionally, patients with a higher percentage reduction in IOP (> 20.94%) had a lower risk of progression compared to those with less reduction. Inadequate IOP reduction, better baseline MD, presence of DH, and lower central corneal thickness were identified as risk factors for progression in advanced OAG patients.

## Introduction

Glaucoma is still a serious public health concern leading to irreversible blindness^1,2^. The clinical burden is greater for patients with advanced glaucoma than for those with early disease, and the rate of decline in quality of life is faster^3^.

Consequently, it is important to identify its long-term prognosis and the factors threatening patients’ visual function. A 5-year prospective, observational study revealed that best-corrected visual acuity (BCVA) at baseline, β-peripapillary atrophy area-to-disc area ratio, and use of systemic antihypertensive agents are significant prognostic factors threatening central visual function in patients with advanced glaucoma^4^. Other studies have demonstrated that severe visual field (VF) loss at diagnosis is a risk factor for progressive severe visual deterioration^4–7^; however, the data on patients with advanced glaucoma is limited.

In this longitudinal observational cohort study spanning more than 10 years, our objective was to examine the long-term outcomes of advanced glaucoma and identify risk factors for progression in patients receiving intraocular pressure (IOP)-lowering treatment. Such knowledge is crucial to proper management of patients with advanced glaucoma, prediction of their functional outcomes, and enhancement of their vision-related quality of life.

## Results

### Demographic and clinical characteristics of study subjects

One hundred and twenty-seven (127) eyes of 127 patients were included in analyses, and 39 patients were excluded. The main reasons for exclusion were a short follow-up period and poor image quality. The baseline characteristics and clinical data on the study participants are summarized in Supplementary Table S1. For all of the included study eyes, the mean follow-up period was 11.37 ± 3.38 years (range, 7.5–15.6 years), and the mean age at diagnosis of advanced glaucoma was 54.06 ± 13.85 years (range, 18–69 years). Among the participants, 68 were male (53.5%) and 59 were female (46.5%). Eleven (11) eyes (8.7%) underwent glaucoma surgery: 10 eyes had trabeculectomy with mitomycin C, and 3 eyes underwent Ahmed drainage device implantation. For 2 eyes, drainage device implantations were secondary procedures after trabeculectomies. Of the 127 eyes, 43 (33.9%) showed structural progression and 54 (42.9%) indicated functional progression. Thirty-six (36) patients (28.3%) demonstrated evidence of both functional and structural progression. Among the patients with structural progression, 7 of 43 eyes exhibited progression on two consecutive spectral-domain optical coherence tomography (SD-OCT) Guided progression analysis (GPA) but not on retinal nerve fiber layer (RNFL) photographs, due to the diffuse type of RNFL thinning.

### Comparison of clinical characteristics of progressors and non-progressors

Of the 127 subjects, 59 eyes (46.5%) showed glaucomatous deterioration and were defined as progressors. Table 1 shows a comparison of the progressors’ and non-progressors’ clinical characteristics. In the progressors, the mean baseline RNFL and ganglion cell-inner plexiform layer (GCIPL) thicknesses were significantly better than those in the non-progressors (65.92 ± 8.65 vs. 61.34 ± 10.15 μm and 63.72 ± 8.04 vs. 60.83 ± 7.63 μm; *P* = 0.007 and *P* = 0.044, respectively). The mean deviation (MD) at baseline also differed between the two groups, with progressors presenting the better values (− 12.77 ± 4.17 vs. − 16.80 ± 5.56 dB; *P* < 0.001). The proportions of patients with underlying hypertension and DH also were higher in progressors (45.8 vs. 23.5% and 12.1 vs. 0%; *P* = 0.008 and *P* = 0.016, respectively). The results of the analysis of the rates of change of the structural and functional parameters are shown in Table 2. The rate of MD change was significantly faster for progressors than for non-progressors (–0.67 ± 0.49 vs. –0.20 ± 0.25 dB/year; *P* < 0.001).

**Table 1:** Comparison between Progressors and Non-progressors .

	Progressors (N = 59)	Non-progressors (N = 68)	*P*-value
Age at first diagnosis of advanced glaucoma (years)	54.25 ± 14.09	53.90 ± 13.73	0.885^a^
Gender (n, %)			
Male	31 (52.5)	37 (54.4)	0.860^b^
Female	28 (47.5)	31 (45.6)	
Diabetes mellitus (n, %)	11 (18.6)	12 (17.6)	0.884^b^
Hypertension (n, %)	27 (45.8)	16 (23.5)	**0.008**^**b**^
History of glaucoma surgery (n, %)	9 (13.2)	2 (3.4)	0.061^b^
Follow-up duration (years)	11.58 ± 3.19	11.28 ± 3.37	0.272^a^
Baseline BCVA (logMAR)	0.04 ± 0.15	0.09 ± 0.20	0.114^a^
Final BCVA (logMAR)	0.29 ± 0.54	0.24 ± 0.38	0.504^a^
Spherical equivalent	− 2.89 ± 4.56	− 2.50 ± 3.84	0.611^a^
Central corneal thickness (μm)	531.49 ± 41.36	524.92 ± 26.44	0.335^a^
Axial length (mm)	25.19 ± 2.34	24.55 ± 1.44	0.094^a^
IOP (mmHg)			
Baseline IOP	16.56 ± 4.34	17.68 ± 5.81	0.218^a^
Mean IOP during follow-up	13.05 ± 1.75	12.87 ± 2.17	0.626^a^
Percentage reduction of IOP (%)	19.36 ± 13.98	21.67 ± 16.88	0.123^a^
IOP fluctuation during follow-up	2.02 ± 0.84	2.18 ± 0.88	0.296^a^
OCT RNFL thickness (μm)			
Baseline average RNFL thickness	65.92 ± 8.65	61.34 ± 10.15	**0.007**^**a**^
Final average RNFL thickness	59.22 ± 7.85	56.89 ± 8.40	0.113^a^
OCT GCIPL thickness (μm)			
Baseline average macular GCIPL thickness	63.72 ± 8.04	60.83 ± 7.63	**0.044**^**a**^
Final average macular GCIPL thickness	58.02 ± 7.88	57.59 ± 8.35	0.771^a^
SAP 24-2 VFI (%)			
Baseline VFI	63.51 ± 14.42	49.88 ± 17.49	** < 0.001**^**a**^
Final VFI	44.69 ± 17.03	44.29 ± 17.44	0.896^a^
SAP 24-2 MD (dB)			
Baseline MD	− 12.77 ± 4.17	− 16.80 ± 5.56	** < 0.001**^**a**^
Final MD	− 19.04 ± 4.73	− 19.09 ± 5.27	0.956^a^
Disc hemorrhage (% positive)	7 (11.9)	1 (1.5)	**0.016**^**b**^

BCVA = best-corrected visual acuity; logMAR = logarithm of the minimum angle of resolution; IOP = intraocular pressure; OCT = optical coherence tomography; RNFL = retinal nerve fiber layer; GCIPL = ganglion cell–inner plexiform layer; SAP = standard automated perimetry; VFI = visual field index; MD = mean deviation.

^a^Student *t* test.

^b^Chi-square test.

Bold values indicate P value reached statistical significance (< 0.05).

**Table 2:** Rates of Change of Structural and Functional Parameters.

	Total	Progressors (N = 59)	Non-progressors (N = 68)	*P*-Value
RNFL thickness parameters (μm/year)				
Average	− 0.52 ± 1.03	− 0.64 ± 1.28	− 0.40 ± 0.71	0.231
Superior	− 0.87 ± 1.55	− 1.17 ± 1.91	− 0.58 ± 1.05	0.055
Inferior	− 0.68 ± 1.13	− 0.77 ± 1.29	− 0.59 ± 0.96	0.406
GCIPL thickness parameters (μm/year)				
Average	− 0.58 ± 0.74	− 0.71 ± 0.91	− 0.46 ± 0.48	0.099
Superior	− 0.60 ± 0.97	− 0.71 ± 1.22	− 0.49 ± 0.62	0.259
Inferior	− 0.55 ± 0.74	− 0.70 ± 0.92	− 0.41 ± 0.49	0.052
Functional parameters				
MD (dB/year)	− 0.43 ± 0.45	− 0.67 ± 0.49	− 0.20 ± 0.25	** < 0.001**
VFI (%/year)	− 1.45 ± 1.63	− 2.35 ± 1.67	− 0.57 ± 0.98	** < 0.001**

Values are mean ± standard deviation.

Bold values indicate *P* value reached statistical significance (< 0.05).

RNFL = retinal nerve fiber layer; GCIPL = ganglion cell–inner plexiform layer; MD = mean deviation; VFI = visual field index.

### Risk factors associated with glaucoma progression

**Figure 1:**
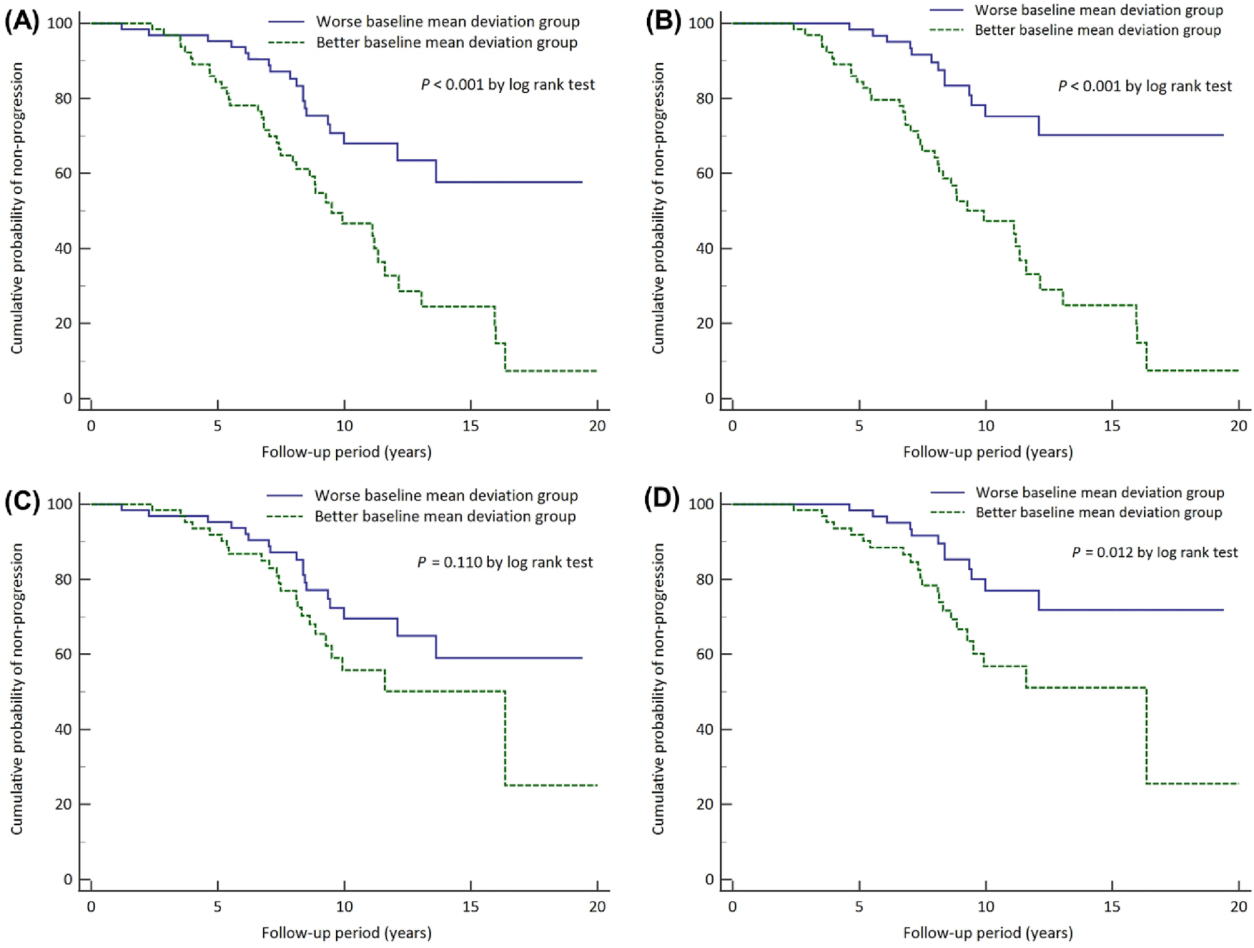
Kaplan–Meier survival plot of glaucoma progression of study population as stratified by baseline mean deviation (MD). The better baseline MD group included those with baseline MD greater than –13.55 dB. The worse baseline MD group (*solid line*) demonstrated a significantly greater cumulative probability of non-progression than did the better baseline MD group (*dotted line*) for (**A**) the structural or functional progression criteria (*P* < 0.001), (**B**) the functional progression criteria (*P* < 0.001), or (**D**) both structural and functional progression criteria (*P* = 0.012). However, for the structural progression criteria, the cumulative probabilities of non-progression between the worse and better baseline MD groups were not significantly different (*P* = 0.110) (**C**).

The results of the univariate and multivariate Cox’s proportional hazard models for the effect of each variable on disease progression are detailed in Table 3. The univariate analysis showed that progression was significantly associated with presence of disc hemorrhage (DH) (*P* = 0.004) and baseline MD (*P* < 0.001). The stepwise multivariate Cox analysis included all of the parameters for which the P-value of the association with VF progression was ≤ 0.1 in the univariate analysis. Glaucoma progression was significantly associated with better baseline VF status (hazard ratios (HR) = 1.109; 95% confidence intervals (CI), 1.044–1.177; *P* = 0.001) and the presence of DH (HR = 2.352; 95% CI, 1.041–5.316; *P* = 0.040). The Kaplan–Meier survival analysis showed that, regardless of whether the progression criteria were structural or functional, only functional, or both, subjects with worse baseline MD had a higher cumulative non-progression probability than did those in the better baseline MD group (log rank test, *P* < 0.001, *P* < 0.001, and *P* = 0.012 for each progression criterion, respectively; Fig. 1 (A,B,D)). However, the analysis was inconsistent only for the structural progression criteria (log rank test, *P* = 0.110; Fig. 1C). In the group with better baseline MD, the lowest baseline MD was −13.55 dB.

**Table 3:** Cox’s proportional hazard models for progression of advanced glaucoma.

	Univariate model			Multivariate model		
	HR	95% CI	*P* Value	HR	95% CI	*P* Value
Demographic data						
Age	1.004	0.985, 1.023	0.662			
Gender, male	0.941	0.562, 1.578	0.819			
Diabetes mellitus	0.992	0.514, 1.914	0.980			
Hypertension	1.546	0.920, 2.597	0.097	1.514	0.899, 2.549	0.119
Clinical data						
IOP						
Baseline IOP	0.957	0.909, 1.007	0.092			
Mean IOP	1.015	0.895, 1.151	0.821			
Percentage reduction of IOP	0.985	0.969, 1.000	0.055	0.993	0.976, 1.011	0.457
IOP fluctuation	0.830	0.596, 1.154	0.266			
Spherical equivalent	0.999	0.935, 1.066	0.967			
Central corneal thickness	0.993	0.984, 1.001	0.105			
Axial length	1.046	0.938, 1.166	0.417			
Disc hemorrhage	3.087	1.390, 6.856	0.004	2.352	1.041, 5.316	0.040
SAP						
Baseline MD	1.125	1.060, 1.194	< 0.001	1.109	1.044, 1.177	0.001
Baseline VFI	1.032	1.016, 1.049	< 0.001			
OCT						
Baseline average RNFL thickness	1.026	1.002, 1.051	0.034			
Baseline average macular GCIPL thickness	1.033	1.001, 1.066	0.040			

Factors with *P* < 0.1 in the univariate analysis were included in the multivariate analysis.

IOP = intraocular pressure; SAP = standard automated perimetry; MD = mean deviation; VFI = visual field index; OCT = optical coherence tomography; RNFL = retinal nerve fiber layer; GCIPL = ganglion cell–inner plexiform layer.

### Comparison of IOP reduction and glaucoma progression

**Figure 2:**
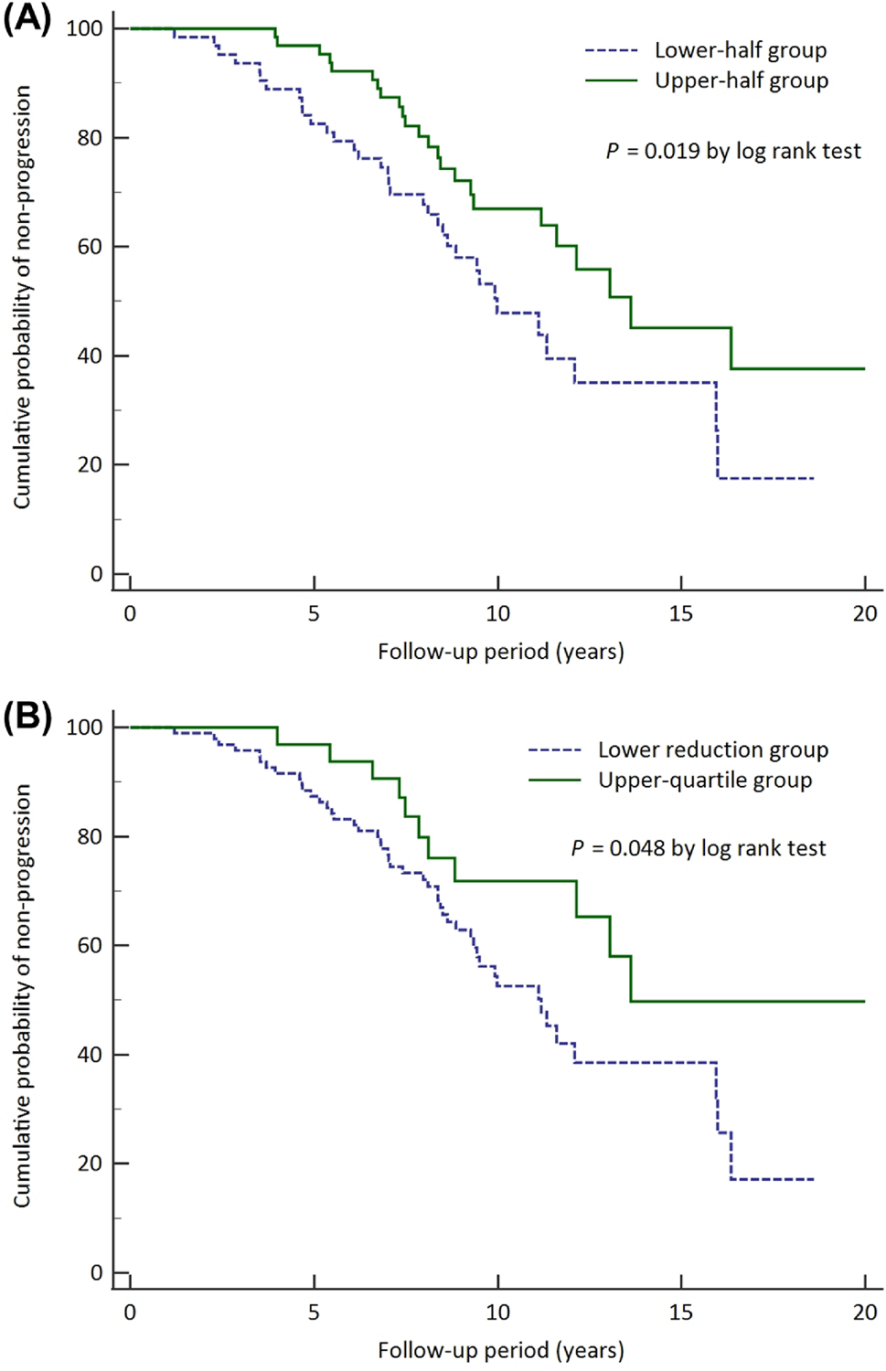
Kaplan–Meier survival plot of glaucoma progression of study population as stratified by percentage reduction of intraocular pressure (IOP). The upper-half group included those with percentage reduction of IOP > 20.9%, and the upper-quartile group included those with percentage reduction of IOP > 31.0%. The cumulative probabilities of non-progression in the upper-half group (*solid line*) and the lower-half group (*dotted line*) were significantly different (*P* = 0.019) (**A**), and the cumulative probabilities of non-progression in the upper-quartile group (*solid line*) and the lower-reduction group (*dotted line*) were significantly different as well (*P* = 0.048) (**B**).

Patients in the upper-half of the percentage-IOP reduction scale had a higher cumulative probability of non-progression to glaucoma than those in the lower-half group, according to a Kaplan–Meier survival analysis (log rank test, *P* = 0.019; Fig. 2A). Supplementary Figure S1 displays Kaplan–Meier survival plots based on both progression criteria and only structural or functional progression criteria (all *P*’s > 0.05). The upper-half group's lowest IOP reduction percentage was 20.94%. The upper-quartile percentage IOP reduction group (lowest IOP reduction of 31.0%) also revealed a higher cumulative probability of non-progression to glaucoma than did those in the lower reduction group, according to a Kaplan–Meier survival analysis (log rank test, *P* = 0.048; Fig. 2B). The Kaplan–Meier survival plots for the groups representative of structural or functional progression alone and both structural and functional progression also were examined. However, the percentage reduction of IOP did not show any significant differences for those progression criteria (all *P*’s > 0.05, Supplementary Figure S2. A multiple regression model revealed significant interactions of percentage-IOP reduction, central corneal thickness (CCT), and baseline standard automated perimetry (SAP) MD with the MD slope (β = 0.014, *P* = 0.003; β = 0.003, *P* = 0.029 and β = –0.029, *P* = 0.002, Table 4).

**Table 4:** Multiple regression analysis for slope of mean deviation.

	Univariate analysis			Multivariate analysis		
	β	95% CI	*P* value	β	95% CI	*P* value
Demographic data						
Age	− 0.004	− 0.010, 0.002	0.174			
Gender, male	− 0.155	− 0.319, 0.009	0.105			
Diabetes mellitus	− 0.046	− 0.259, 0.168	0.673			
Hypertension	− 0.085	− 0.260, 0.090	0.337			
Clinical data						
IOP						
Baseline IOP	0.019	0.004, 0.034	0.015	− 0.027	− 0.061, 0.003	0.075
Mean IOP	− 0.004	− 0.046, 0.038	0.850			
Percentage reduction of IOP	0.008	0.003, 0.013	0.001	0.014	0.005, 0.024	0.003
IOP fluctuation	0.111	0.019, 0.203	0.019	0.038	− 0.090, 0.167	0.554
Spherical equivalent	− 0.016	− 0.036, 0.004	0.117			
Central corneal thickness	0.003	0.001, 0.006	0.011	0.003	0.000, 0.005	0.029
Axial length	0.011	− 0.034, 0.057	0.501			
Disc hemorrhage	− 0.261	− 0.589, 0.066	0.116			
Baseline BCVA	0.481	0.030, 0.932	0.037	0.189	− 0.286, 0.664	0.432
SAP						
Baseline MD	− 0.035	− 0.050, − 0.020	< 0.001	− 0.029	− 0.047, − 0.011	0.002
Baseline VFI	− 0.010	− 0.015, − 0.006	< 0.001			
OCT						
Baseline average RNFL thickness	− 0.003	− 0.012, 0.005	0.472			
Baseline average macular GCIPL thickness	− 0.011	− 0.022, − 0.001	0.036	− 0.007	− 0.017, 0.004	0.220

Factors with *P* < 0.1 in the univariate analysis were included in the multivariate analysis.

IOP = intraocular pressure; SAP = standard automated perimetry; MD = mean deviation; VFI = visual field index; OCT = optical coherence tomography; RNFL = retinal nerve fiber layer; GCIPL = ganglion cell–inner plexiform layer.

### Comparison of clinical characteristics of participants with and without low vision

Nineteen (19) eyes (15.0%) of patients exhibited low vision at the most recent follow-up. When comparing the clinical characteristics between the patients presenting low vision at the final visit and those not, the baseline BCVA among patients with low vision was 0.24 ± 0.30 logMAR, which was worse than that among patients without low vision (0.04 ± 0.13 logMAR; *P* < 0.001), while baseline average GCIPL thickness was significantly lower (58.11 ± 7.94 vs. 62.94 ± 7.73 μm; *P* < 0.022, Supplementary Table S2). As for IOP fluctuation, it was significantly greater in the low-vision group (2.49 ± 1.12 vs. 2.03 ± 0.80; *P* = 0.034).

### Risk factors associated with low vision

Medical history of hypertension, IOP fluctuation and baseline BCVA were found to be associated with visual impairment by the univariate Cox’s proportional hazard model (Supplementary Table S3). In the multivariate Cox’s proportional regression analysis employing a forward conditional method, poor baseline BCVA was significantly associated with presentation of low vision (HR = 8.915; 95% CI, 1.378–57.666; *P* = 0.022).

### Representative cases

**Figure 3:**
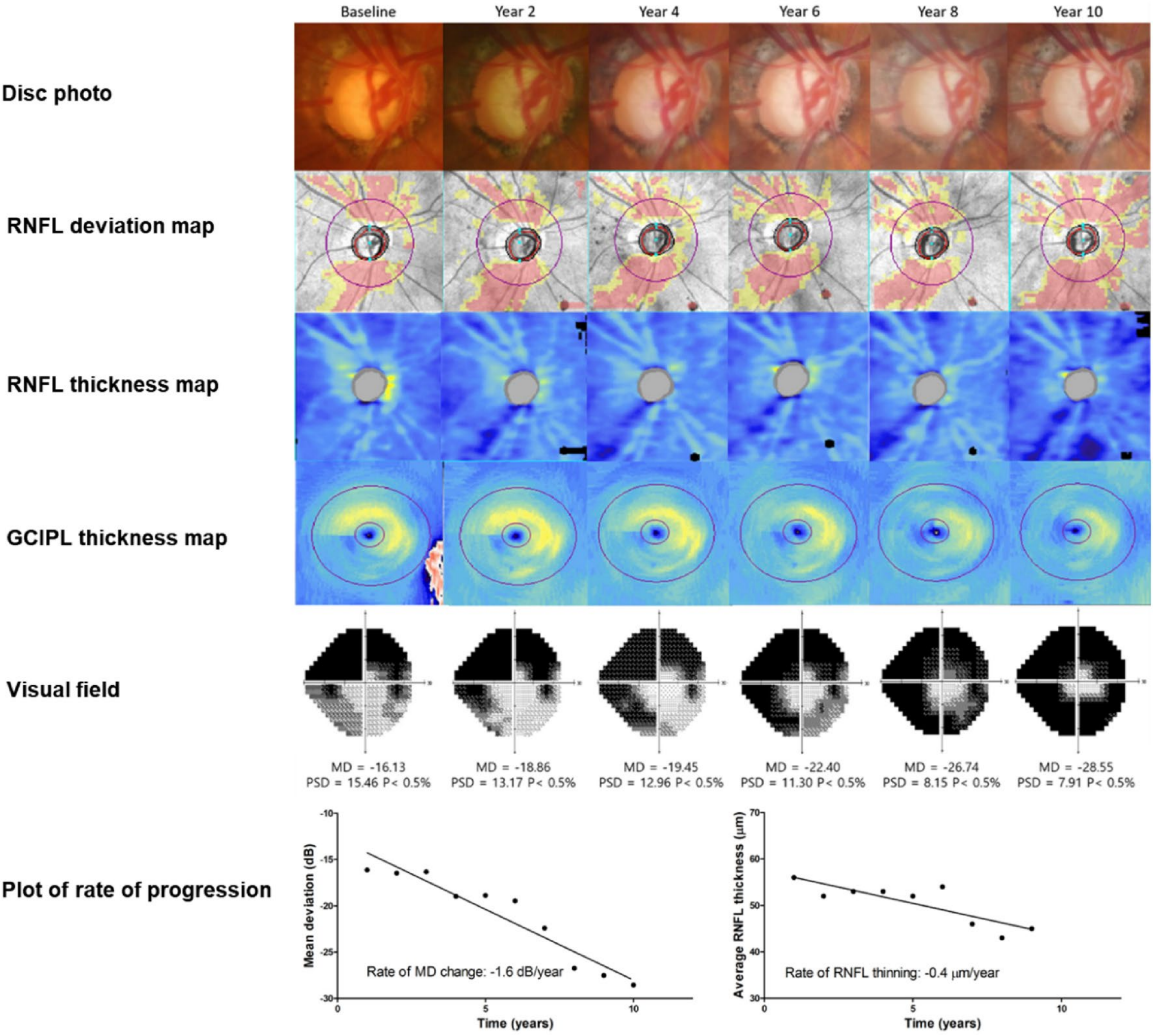
Representative case of progression of patient with advanced glaucoma. After a follow-up of 10 years, a male patient (age: 69) showed VF progression of advanced glaucoma with an MD change of − 1.60 dB/year and an average RNFL thickness change of –0.40 μm/year.

**Figure 4:**
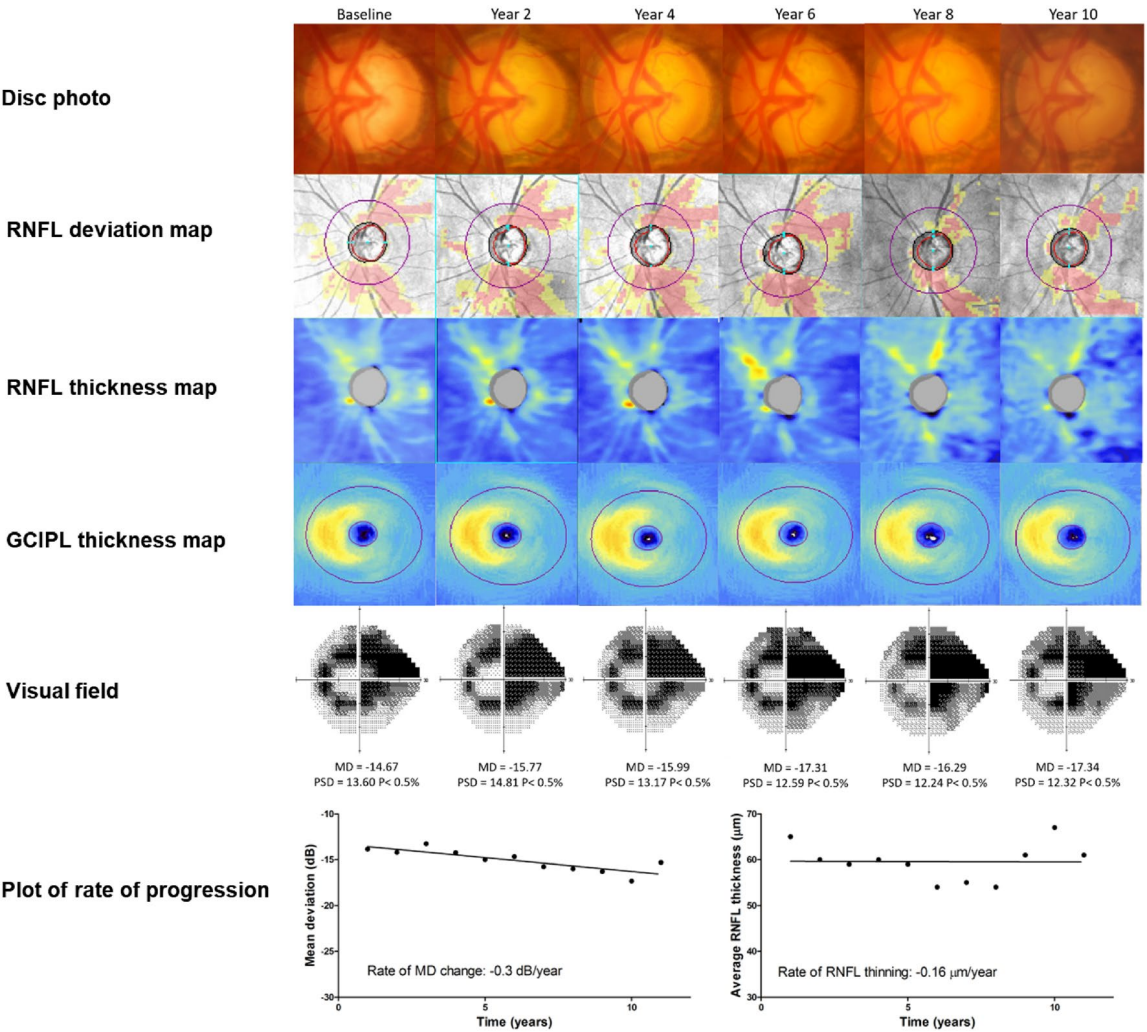
Representative case of non-progression of patient with advanced glaucoma. After a follow-up 11 years, a male patient (age: 66) manifested no signs of glaucoma progression. The mean MD change was –0.30 dB/year and the average RNFL thickness change was –0.16 μm/year.

Representative cases of patients with advanced glaucoma are shown in Fig. 3 and 4. Figure 3 shows the longitudinal data for a male patient (age: 69) who demonstrated VF progression at the MD change of − 1.60 dB/year and the average RNFL thickness slope of −0.40 μm/year after 10 years of follow-up. Figure 4 displays the results for a male patient (age: 66) who demonstrated no signs of glaucoma progression during the 11-year follow-up period.

## Discussion

This longitudinal cohort study investigated the clinical course of advanced- open-angle glaucoma (OAG) patients and assessed the progression rate and risk factors of glaucoma progression for them. During a follow-up period of 10 years or more, 46.5% of patients showed glaucoma progression with a mean MD change of − 0.67 ± 0.49 dB/year. Better baseline VF status, presence of DH, inadequate IOP lowering, and lower CCT were factors associated with glaucoma progression.

There have been previous attempts to demonstrate the clinical course and risk factors for advanced glaucoma. The Advanced Glaucoma Intervention Study (AGIS), the first multicenter randomized clinical trial concerning advanced glaucoma^8^, demonstrated that IOP-lowering extent was associated with progression of VF damage over an 8-year period^9^. Kim et al.^10^ retrospectively reviewed 87 eyes of medically treated advanced- normal tension glaucoma (NTG) patients with a baseline MD − 16.6 dB, and reported that 62.2% of them showed progression of VF damage over a period of 5.3 years. In their study, younger age and lower initial BCVA were found to be significant risk factors for loss of visual function. Recently, the Treatment of Advanced Glaucoma Study identified newly diagnosed advanced-glaucoma patients over the course of 36 months of follow-up at 27 centers^11^. The trial found no evidence of any difference in vision-related quality of life between trabeculectomy or glaucoma drops^12^; however, trabeculectomy significantly reduced the proportion of progressing eyes at 24 months^13^. Although clinical course is widely studied, to our knowledge, longitudinal investigations of 10 years’ duration or longer are limited. In this study, we analyzed advanced glaucoma over the course of a mean follow-up period of 11 years. We believe that the long-term results of our study could offer deeper insight into end-stage glaucoma.

The necessity of reducing IOP for treatment of glaucoma has been convincingly proven by previous investigations^14–16^. The AGIS results showed that patients who maintained an IOP of less than 18 mmHg over the course of six years had no VF progression^17^. According to our own findings (Fig. 2), the group that met the treatment goal of 20% IOP reduction was observed to have lesser glaucoma progression. However, the majority of the differences in Kaplan–Meier survival plots seem to have happened early, and thereafter the slopes parallelized. Long-term improvements in IOP control may lessen the impact of these early significant disparities.

In the present study, occurrence of DH during the study period significantly increased the risk of glaucoma progression. Usually, DH is thought to be frequently observed for the early to moderate stage of glaucoma and to show decreased incidence in optic discs with advanced cupping, especially in regions where the neuroretinal rim is absent^18,19^. The results of this study emphasize that the presence of DH is of vital importance, even for detection of progression of advanced glaucoma. Our results are consistent with previous studies in this regard^19–23^. In fact, development of DH may be linked to progression at all stages of glaucoma. Since progressors in the current study included a high percentage of patients with hypertension, some DH development may be associated with vascular factors. Furthermore, the inconsistency of the factors identified by the multiple regression model and Cox's proportional hazard models should be taken into account when considering the findings of our study. Different endpoint criteria might have contributed to the inconsistent results.

This study demonstrated that subjects with less severe VF defect at baseline were more likely to show glaucoma progression during follow-up. However, the relationship between baseline VF status and the possibility of glaucoma progression remains controversial. Several previous studies’ results are consistent with ours in showing that better baseline VF status increases the risk of progression^9,24^. On the other hand, some other studies determined, contradictorily, that severe VF loss at baseline is a risk factor for progressive severe visual deterioration^7,25–27^. A possible explanation for this discrepancy may be related to differences in study populations. All of our participants were classified as advanced glaucoma according to the Hodapp-Parrish-Anderson criteria, based on a mean baseline SAP MD of − 14.93 dB, which, compared with participants in other previous studies, did not represent severe damage^4^. Additionally, the "floor effect" of OCT measurements might be another reason contributing to the under-detection of disease progression for the individuals with severely damaged glaucoma. Further study will be necessary to elucidate the complex relationships between baseline severity of glaucoma and progression of VF damage.

Detecting progression of advanced glaucoma, which is an essential part of glaucoma management, is challenging due to the poor accuracy of structural thickness measurements^28^. The “floor effect” is defined as the point at which no further structural loss can be detected, and this, in fact, is thought to be a serious problem for monitoring of structural deterioration^28,29^. In this study, OCT was ineffective in detecting progression for those who had worse baseline MD (Fig. 1C). There have been attempts made to estimate SD-OCT-measured average floors for RNFL thickness. Bowd et al.^30^ estimated the measurement floor for structural analysis based on variability in stable glaucomatous eyes using an arbitrary variability cutoff, and demonstrated that the average floors were 38 μm for circumpapillary RNFL thickness and 38 μm for GCIPL thickness. According to other previous reports, the estimated average RNFL thickness measurement floor ranged from 44.9 to 53.7 μm^31–34^. In the present study, the baseline average RNFL and GCIPL thicknesses averaged 63.48 and 62.20 μm, and the average RNFL and GCIPL thicknesses at final visit were measured to 57.99 and 57.79 μm, respectively (Supplementary Table S1). The mean RNFL and GCIPL thicknesses tended to be greater than the estimated measurements of previous studies, suggesting that the eyes enrolled for the present study had not reached the floor. This might have been due to the fact that these glaucomatous eyes were less advanced at baseline than were those in the relevant previous investigations.

This study has several limitations which should be considered when interpreting our results. First, the medical records reviewed were from a single tertiary medical center, and so selection bias might have been operative. The inclusion of 11 patients (8.7%) who had undergone glaucoma surgery could have biased our results. Our results may also have been affected by the exclusion of 8 patients (6.3%) who did not have 5 reliable results from VF tests. Second, all of the participants in this study were Koreans. Racial differences may have contributed to the study subjects’ comparatively younger age of glaucoma onset. Longitudinal studies including non-East Asian patients can afford further clarification and more comprehensive knowledge of advanced glaucoma. Lastly, all of the study participants were categorized as having advanced glaucoma, with a mean baseline SAP MD of − 14.13 dB. Consequently, there appears to be insufficient data available on patients with even more severe advanced glaucoma.

In conclusion, this study presents data on eyes with advanced glaucoma that had been followed during a mean period of 11 years. Our findings suggest that development of DH, better baseline VF status and inadequate IOP control are significant risk factors for advanced-OAG progression. This should be considered in monitoring patients with advanced glaucoma.

## Methods

This study included subjects from an ongoing study’s OAG and NTG cohort at the Glaucoma Clinic of Seoul National University Hospital, and the study protocol was approved and informed consents were waived due to the retrospective nature of this study by the Institutional Review Board (IRB) of Seoul National University Hospital (IRB no. 2304-077-1422). All of the investigations adhered to the tenets of the Declaration of Helsinki.

### Study participants

In this longitudinal cohort study, we reviewed the medical records of patients who had been diagnosed with advanced OAG and followed up for more than 7 years. Advanced glaucoma is classified according to the ‘severe’ category of VF loss based on the Hodapp-Parrish-Anderson criteria^35^: (1) MD worse than − 12 dB, (2) at least 50% of the points depressed at *P*-value < 5 or 20% of the points at *P*-value < 1% in the pattern deviation plot, (3) at least 1 point in the central 5 degrees with a sensitivity of 0 dB, or (4) points within the central 5 degrees with sensitivity < 15 dB in both hemifields.

We initially selected 166 patients who had visited Seoul National University Glaucoma Clinic from August 2001 to September 2015 and been diagnosed with advanced OAG. Of those, patients who met the following criteria were enrolled: (1) treatment and a follow-up period of at least 7 years after the diagnosis of advanced OAG and (2) at least 5 reliable SAP results acquired with the Humphrey Field Analyzer (Carl Zeiss Meditec, Dublin, CA, USA) with the 24–2 test pattern using the Swedish Interactive Thresholding Algorithm (SITA) standard test protocol. VF results with fixation losses < 20%, false negatives ≤ 15%, and false positives ≤ 15% were included as reliable VFs. The exclusion criteria were as follows: (1) any history of ophthalmic surgery (except uncomplicated cataract surgery and glaucoma surgery) or retinal laser photocoagulation; (2) occurrence of ophthalmic disease other than glaucoma that might affect visual function; (3) presence of pseudoexfoliation or pigment dispersion syndrome; (4) possibility of secondary glaucoma related to uveitis, and (5) poor image quality. A total of 127 subjects were included in the evaluation. If both eyes were eligible for the investigation, one eye was selected by computerized random selection.

Based on the most acknowledged and tolerated medical therapy, it was the goal for every patient to reduce IOP by at least 20% and to less than 18 mmHg. In cases where this was not accomplished, surgical interventions were combined with medication therapy to lower IOP.

### Clinical and ophthalmic evaluations

All of the study participants underwent complete ophthalmic examinations including measurement of BCVA, refractive error, slit-lamp biomicroscopy, IOP measurements using the Goldmann applanation tonometer (Haag-Streit, Konig, Switzerland), gonioscopy, dilated funduscopic examination, CCT measurement (Pocket II Pachymeter Echo Graph; Quantel Medical, Clermont-Ferrand, France), axial length (AXL) measurement (Axis II PR; Quantel Medical, Inc., Bozeman, MT, USA), stereoscopic optic disc photography, red-free RNFL photography (TRC-50IX; Topcon Corporation, Tokyo, Japan), as well as optic nerve head and macular imaging by Cirrus SD-OCT (Carl Zeiss Meditec), and SAP 24-2 testing (Humphrey Field Analyzer; Carl Zeiss Meditec) according to the SITA standard strategy. SD-OCT and SAP 24-2 testing were performed annually. Fluctuation of IOP was defined as a standard deviation of IOP during the follow-up period, and percentage reduction of IOP was determined using the following formula 100 × (baseline IOP—mean IOP during follow-up)/baseline IOP. The World Health Organization (WHO) classification was used to determine the level of visual impairment as measured by visual acuity. According to the WHO standards, blindness was classified as BCVA worse than 3/60 or constriction of the central VF to less than 10 degrees and severe visual impairment as BCVA between 6/60 and 3/60. In this study, participants who had BCVA worse than 6/60 were defined as low vision based on VA, and were designated as being in the low-vision group.

### Assessment of optic disc hemorrhage

DH was defined as an isolated hemorrhage located in the disc tissue, or in the peripapillary retina connected to the disc rim, that is not associated with optic disc edema, papillitis, diabetic retinopathy, central or branch retinal vein occlusion, or any other retinal disease^36^. Throughout the study, occurrence of DH was evaluated at every visit by two glaucoma specialists (Y.I.S. and J.W.J.) based on stereoscopic optic disc photography and red-free RNFL photography. Discrepancies between the two observers’ findings were resolved by discussion and consensus.

### SD-OCT imaging for evaluation of structural progression

The RNFL and GCIPL thicknesses were measured using Cirrus SD-OCT version 9.5 software (Carl Zeiss Meditec). Optic disc scans (Optic Disc Cube 200 × 200 protocol) and macular scans (Macular Cube 512/128 protocol) were performed. Peripapillary RNFL thickness was verified using a measurement circle with a diameter of 3.46 mm, and macular GCIPL thickness was measured within an annulus-shaped measurement area with internal vertical and horizontal diameters of 1 and 1.2 mm, respectively, and external vertical and horizontal diameters of 4 and 4.8 mm, respectively. The Cirrus SD-OCT GPA software program determined RNFL and GCIPL progression using information from the optic disc and macular scans. Trend analysis was performed on the average RNFL and GCIPL thicknesses by linear regression between average RNFL/GCIPL thickness and age. Event analysis was performed on the RNFL thickness profiles and maps. “Possible loss” was determined when the differences between the 2 baseline and follow-up measurements surpassed the test–retest variability, and “likely loss” was established when the “possible loss” condition was satisfied on 2 consecutive visits.

Well-focused and high-quality images with a signal strength of at least 6 were included in the analyses, while low-quality images exhibiting motion artifacts, segmentation errors or inadequate centration were eliminated.

## Determination of glaucoma progression

Glaucomatous VF damage was defined as (1) glaucoma hemifield test values outside the normal limits or (2) three or more abnormal contiguous points with a probability of *P* < 0.05, of which at least one point has a probability of *P* < 0.01 on a pattern deviation plot, or (3) a pattern standard deviation of *P* < 0.05. All of the participants received treatment with one or more topical medications according to the decisions of the glaucoma specialists in each case.

Glaucoma progression was defined as either structural alterations on optic disc and/or RNFL or functional alterations on VF tests. When “likely loss” or “possible loss” on at least two successive tests was discovered in the SD-OCT GPA event-based analysis and the change was also confirmed on the most recent optic disc and/or RNFL photography, the condition was classified as structural progression. Functional progression was determined by event-based analysis using the GPA software of the Humphrey Field Analyzer. It was defined as a significant decrease from the baseline (initial two reliable VF tests) pattern deviation at three or more of the same test points on two or three consecutive VF tests, which is classified by the software as “possible progression” or “likely progression,” respectively^37^. Using the trend-based analysis of the Humphrey Field Analyzer, we assessed the rate of glaucoma progression in accordance with the change of MD against time.

### Data analysis

The data of the study subjects were analyzed using IBM SPSS software (version 25.0; IBM Corporation, Armonk, NY, USA) and were expressed as mean ± standard deviations or numbers (%). The progressors and non-progressors as well as those with and without low vision were compared using independent 2-sample t tests or Mann–Whitney U tests for continuous variables and Chi-square tests or Fisher’s exact tests for categorical variables. Factors related to glaucoma progression and incidence of low vision were analyzed using the univariate and multivariate Cox proportional hazards models; HR and 95% CI were reported. The cumulative probability of glaucoma non-progression in the upper- and lower-half subgroups stratified by percentage IOP reduction was analyzed and compared by Kaplan–Meier survival analysis. The log-rank test was performed to compare the groups with upper- and lower-half percentage reduction of IOP. The upper-quartile and lower percentage reduction groups (including the lower-three-quartile groups) were compared in the same way. Based on the baseline MD of each patient, the entire study population was divided into two subgroups: the worse and better baseline MD groups. Then, the cumulative probability of glaucoma non-progression in those two groupings was evaluated and compared by Kaplan–Meier survival analysis and log-rank testing. The MD slope magnitude was examined using multiple linear regression models, and coefficients (β) with 95% Cis were derived after adjusting for additional clinical variables. All of the calculated P-values were two-sided, and *P* < 0.05 was considered to be statistically significant.

### Supplementary Information

Supplementary Information 1.

Supplementary Information 2.

Supplementary Information 3.

Supplementary Information 4.

Supplementary Information 5.

Supplementary Information 6.

Supplementary Information 7.

## Data Availability

The corresponding author will provide the study's data upon reasonable request.

## Abbreviations

BCVA: Best-corrected visual acuity
VF: Visual field
IOP: Intraocular pressure
SD-OCT: Spectral domain optical coherence tomography
GPA: Guided progression analysis
RNFL: Retinal nerve fiber layer
GCIPL: Ganglion cell-inner plexiform layer
MD: Mean deviation
DH: Disc hemorrhage
HR: Hazard ratios
CI: Confidence intervals
CCT: Central corneal thickness
SAP: Standard automated perimetry
OAG: Open-angle glaucoma
AGIS: Advanced glaucoma intervention study
NTG: Normal tension glaucoma
IRB: Institutional Review Board
SITA: Swedish Interactive Thresholding Algorithm
AXL: Axial length
WHO: World Health Organization
