# Supplementary figures and images for: Longitudinal evaluation of advanced glaucoma: ten year follow-up cohort study
Source: Sci Rep. 2024 Jan 4;14:476. doi: 10.1038/s41598-023-50512-7 (PMC10766632; doi:10.1038/s41598-023-50512-7)

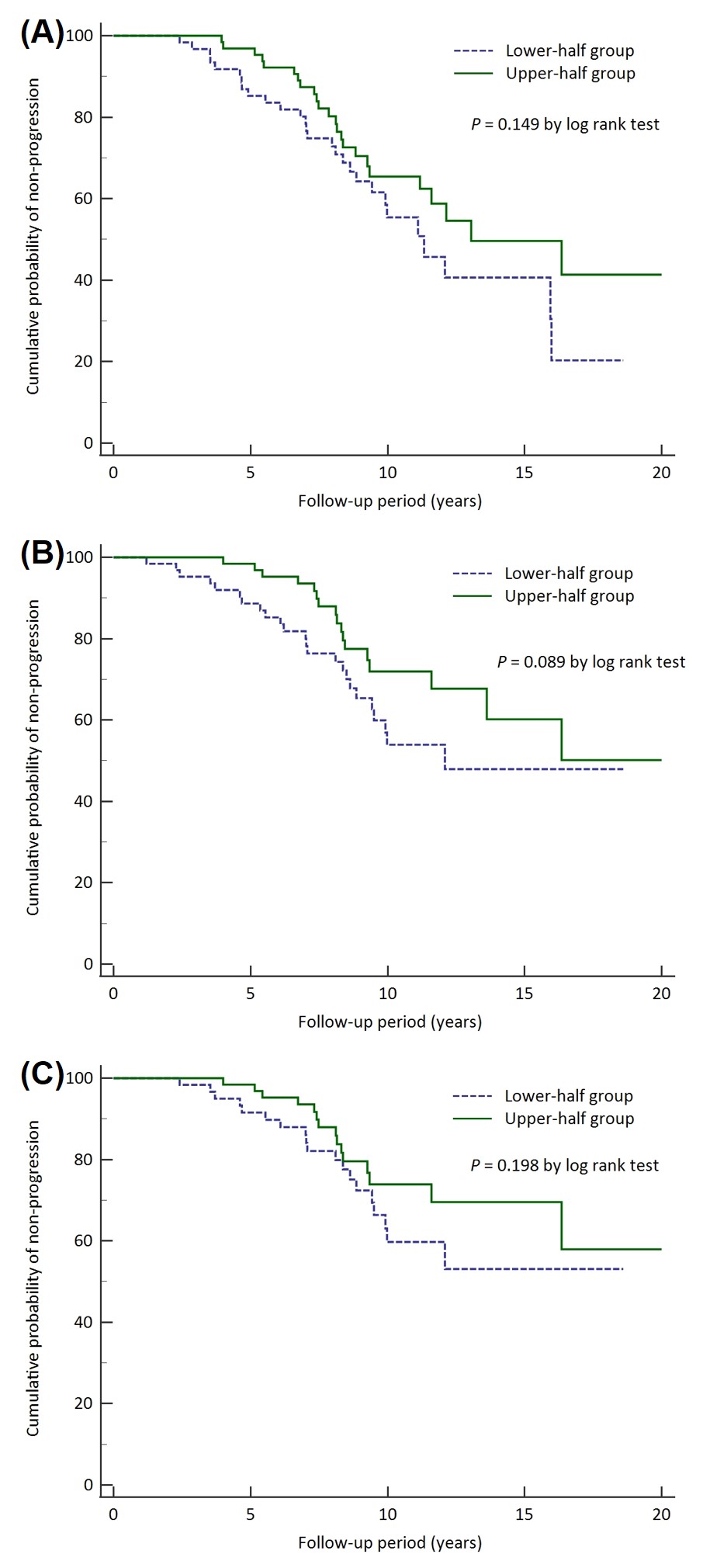

Supplement: Supplementary file 2 — Supplementary Information 2. [file 41598_2023_50512_MOESM2_ESM.jpg]

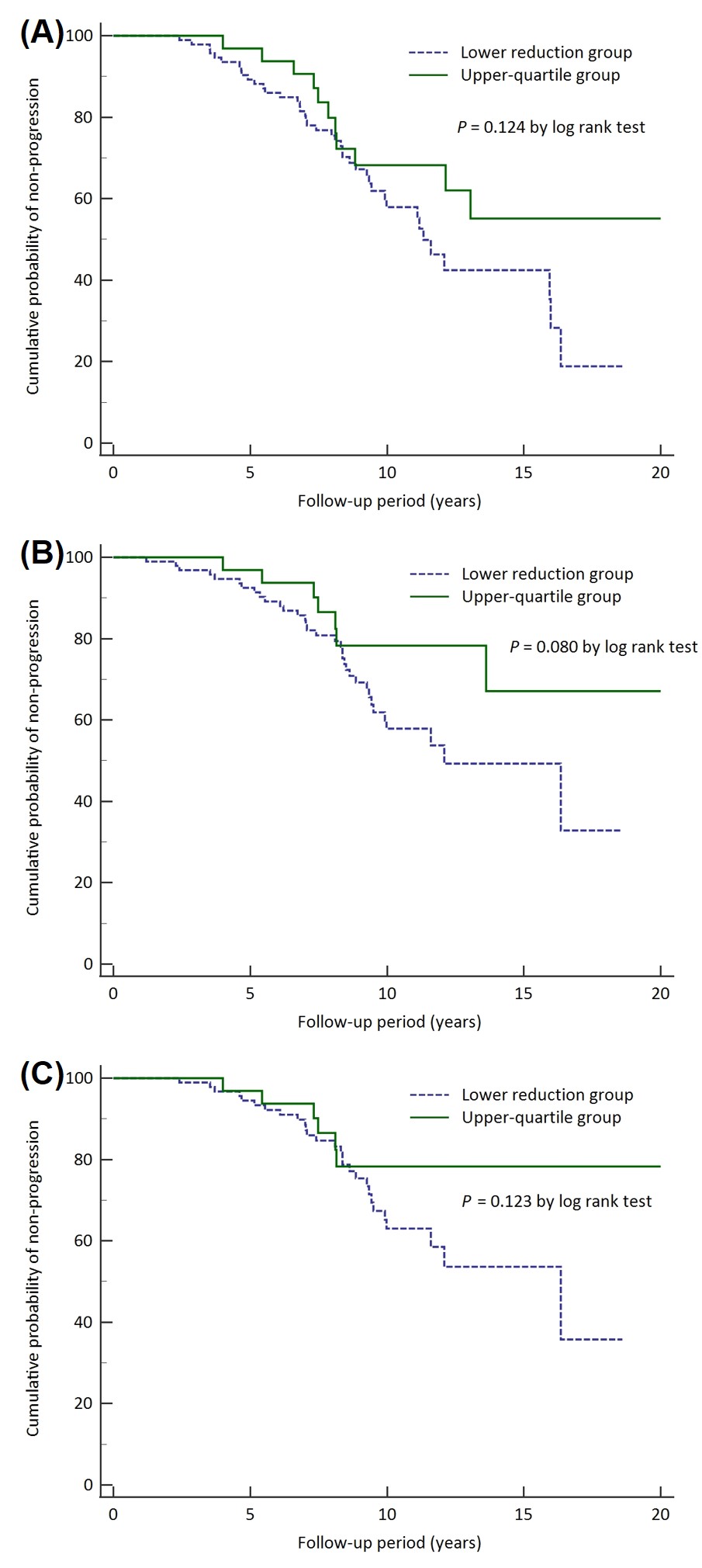

Supplement: Supplementary file 4 — Supplementary Information 4. [file 41598_2023_50512_MOESM4_ESM.jpg]
