# Supplementary material for: Longitudinal evaluation of advanced glaucoma: ten year follow-up cohort study
Source: Sci Rep. 2024 Jan 4;14:476. doi: 10.1038/s41598-023-50512-7 (PMC10766632; doi:10.1038/s41598-023-50512-7)
Supplement: Supplementary file 3 — Supplementary Information 3. [file 41598_2023_50512_MOESM3_ESM.docx]

**Supplementary Figure 2. Kaplan–Meier survival plot of glaucoma progression of study population as stratified by percentage reduction of intraocular pressure (IOP).** The upper-quartile group included those with percentage reduction of IOP > 31.0%. The cumulative probabilities of non-progression in the upper-quartile group (solid line) and lower-reduction group (dotted line) were not significantly different for (A) the functional progression criteria (*P*=0.124), (B) the structural progression criteria (*P*=0.080) or (C) both structural and functional progression criteria (*P*=0.123).
