# Supplementary material for: Longitudinal evaluation of advanced glaucoma: ten year follow-up cohort study
Source: Sci Rep. 2024 Jan 4;14:476. doi: 10.1038/s41598-023-50512-7 (PMC10766632; doi:10.1038/s41598-023-50512-7)
Supplement: Supplementary file 5 — Supplementary Information 5. [file 41598_2023_50512_MOESM5_ESM.docx]

**Supplementary Table 1. Participant Characteristics**

| Characteristics | Data (N=127) |
| --- | --- |
| Age at first diagnosis of advanced glaucoma (years) | 54.06 ± 13.85 |
| Gender (n, %) |  |
| Male | 68 (53.5) |
| Female | 59 (46.5) |
| Diabetes mellitus (n, %) | 23 (18.1) |
| Hypertension (n, %) | 43 (33.9) |
| Type of glaucoma (n, %) |  |
| HTG | 37 (29.1) |
| NTG | 90 (70.9) |
| History of glaucoma surgery (n, %) | 11 (8.7) |
| Follow-up duration (years) | 11.37 ± 3.38 |
| BCVA at baseline (logMAR) | 0.07 ± 0.18 |
| Spherical equivalent | −2.67 ± 4.16 |
| Central corneal thickness (μm) | 528.48 ± 35.31 |
| Axial length (mm) | 24.84 ± 1.91 |
| IOP (mmHg) |  |
| Baseline IOP | 17.16 ± 5.19 |
| Mean IOP during follow-up | 12.95 ± 1.98 |
| Percentage reduction of IOP (%) | 20.67 ± 15.69 |
| IOP fluctuation during follow-up | 2.10 ± 0.87 |
| OCT RNFL thickness (μm) |  |
| Baseline average RNFL thickness | 63.48 ± 9.71 |
| Final average RNFL thickness | 57.99 ± 8.20 |
| OCT GCIPL thickness (μm) |  |
| Baseline average macular GCIPL thickness | 62.20 ± 7.93 |
| Final average macular GCIPL thickness | 57.79 ± 8.10 |
| SAP 24-2 VFI (%) |  |
| Baseline VFI | 56.21 ± 17.46 |
| Final VFI | 44.48 ± 17.18 |
| SAP 24-2 MD (dB) |  |
| Baseline MD | −14.93 ± 5.33 |
| Final MD | −19.07 ± 5.01 |
| Disc hemorrhage (n, %) | 8 (6.3) |

HTG = high tension glaucoma; NTG = normal tension glaucoma; BCVA = best-corrected visual acuity; logMAR = logarithm of the minimum angle of resolution; IOP = intraocular pressure; OCT = optical coherence tomography; RNFL = retinal nerve fiber layer; GCIPL = ganglion cell–inner plexiform layer; SAP = standard automated perimetry; VFI = visual field index; MD = mean deviation
