# Supplementary material for: Longitudinal evaluation of advanced glaucoma: ten year follow-up cohort study
Source: Sci Rep. 2024 Jan 4;14:476. doi: 10.1038/s41598-023-50512-7 (PMC10766632; doi:10.1038/s41598-023-50512-7)
Supplement: Supplementary file 6 — Supplementary Information 6. [file 41598_2023_50512_MOESM6_ESM.docx]

**Supplementary Table 2. Comparison between Participants With and Without Low Vision**

|  | With Low Vision (N=19) | Without low vision (N=108) | P-Value |
| --- | --- | --- | --- |
| Age (years) | 53.21 ± 12.45 | 54.21 ± 14.13 | 0.754 ^a^ |
| Gender (n, %) |  |  |  |
| Male | 9 (47.4) | 59 (54.6) | 0.623 ^b^ |
| Female | 10 (52.6) | 49 (45.4) |  |
| Diabetes mellitus (n, %) | 2 (10.5) | 21 (19.4) | 0.523 ^b^ |
| Hypertension (n, %) | 4 (21.1) | 39 (36.1) | 0.294 ^b^ |
| History of glaucoma surgery (n, %) | 4 (21.1) | 7 (6.5) | 0.060 ^c^ |
| Follow-up duration (years) | 13.37 ± 3.87 | 11.02 ± 3.17 | 0.170 ^a^ |
| Baseline BCVA (logMAR) | 0.24 ± 0.30 | 0.04 ± 0.13 | **<0.001 ^a^** |
| Final BCVA (logMAR) | 1.08 ± 0.69 | 0.12 ± 0.17 | **<0.001 ^a^** |
| Spherical equivalent | −4.47 ± 5.74 | −2.34 ± 3.74 | 0.135 ^a^ |
| Central corneal thickness (μm) | 530.31 ± 57.32 | 528.16 ± 30.45 | 0.823 ^a^ |
| Axial length (mm) | 25.77 ± 3.08 | 24.68 ± 1.61 | 0.187 ^a^ |
| IOP (mmHg) |  |  |  |
| Baseline IOP | 19.58 ± 7.46 | 16.73 ± 4.60 | 0.122 ^a^ |
| Mean IOP during follow-up | 13.67 ± 1.89 | 12.83 ± 1.98 | 0.089 ^a^ |
| Percentage reduction of IOP | 23.33 ± 20.45 | 20.20 ± 14.77 | 0.425 ^a^ |
| IOP fluctuation during follow-up | 2.49 ± 1.12 | 2.03 ± 0.80 | **0.034 ^a^** |
| OCT RNFL thickness (μm) |  |  |  |
| Baseline average RNFL thickness | 62.21 ± 8.82 | 63.71 ± 9.88 | 0.509 ^a^ |
| Final average RNFL thickness | 57.00 ± 6.86 | 58.17 ± 8.43 | 0.515 ^a^ |
| OCT GCIPL thickness (μm) |  |  |  |
| Baseline average macular GCIPL thickness | 58.11 ± 7.94 | 62.94 ± 7.73 | **0.022 ^a^** |
| Final average macular GCIPL thickness | 54.32 ± 9.71 | 58.42 ± 7.66 | 0.095 ^a^ |
| SAP 24-2 VFI (%) |  |  |  |
| Baseline VFI | 53.26 ± 20.21 | 56.73 ± 16.98 | 0.488 ^a^ |
| Final VFI | 37.16 ± 19.92 | 45.77 ± 16.42 | 0.088 ^a^ |
| SAP 24-2 MD (dB) |  |  |  |
| Baseline MD | −15.65 ± 6.64 | −14.80 ± 5.10 | 0.601 ^a^ |
| Final MD | −20.37 ± 6.04 | −18.84 ± 4.80 | 0.306 ^a^ |
| Rate of SAP MD change (dB/year) | −0.55 ± 0.64 | −0.41 ± 0.42 | 0.258 ^a^ |
| Disc hemorrhage (% positive) | 0 (0%) | 8 (7.4%) | 0.361 ^b^ |

BCVA = best-corrected visual acuity; logMAR = logarithm of the minimum angle of resolution; IOP = intraocular pressure; OCT = optical coherence tomography; RNFL = retinal nerve fiber layer; GCIPL = ganglion cell–inner plexiform layer; SAP = standard automated perimetry; VFI = visual field index; MD = mean deviation

^a^ Mann-Whitney U test

^b^ Chi-square test

^c^ Fisher’s exact test

Bold values indicate P value reached statistical significance (<0.05)
