# Supplementary material for: Longitudinal evaluation of advanced glaucoma: ten year follow-up cohort study
Source: Sci Rep. 2024 Jan 4;14:476. doi: 10.1038/s41598-023-50512-7 (PMC10766632; doi:10.1038/s41598-023-50512-7)
Supplement: Supplementary file 7 — Supplementary Information 7. [file 41598_2023_50512_MOESM7_ESM.docx]

**Supplementary Table 3. Cox Proportional Hazard Models for Low Vision**

|  | Univariate model | | | Multivariate model | | |
| --- | --- | --- | --- | --- | --- | --- |
|  | HR | 95% CI | P Value | HR | 95% CI | P Value |
| Demographic data |  |  |  |  |  |  |
| Age | 1.004 | 0.971, 1.039 | 0.795 |  |  |  |
| Gender, male | 1.594 | 0.642, 3.956 | 0.315 |  |  |  |
| Diabetes mellitus | 0.600 | 0.137, 2.629 | 0.498 |  |  |  |
| Hypertension | 0.304 | 0.098, 0.949 | 0.040 | 0.366 | 0.115, 1.167 | 0.089 |
| Clinical data |  |  |  |  |  |  |
| IOP |  |  |  |  |  |  |
| Baseline IOP | 1.057 | 0.990, 1.129 | 0.098 |  |  |  |
| Mean IOP | 1.195 | 0.940, 1.518 | 0.146 |  |  |  |
| Percentage reduction of IOP | 1.008 | 0.982, 1.035 | 0.547 |  |  |  |
| IOP fluctuation | 1.533 | 1.021, 2.301 | 0.039 | 1.061 | 0.600, 1.875 | 0.840 |
| Spherical equivalent | 0.938 | 0.853, 1.032 | 0.189 |  |  |  |
| Central corneal thickness | 0.993 | 0.978, 1.009 | 0.403 |  |  |  |
| Axial length | 1.068 | 0.913, 1.249 | 0.411 |  |  |  |
| Disc hemorrhage | 0.043 | 0.001, 64.201 | 0.399 |  |  |  |
| Baseline BCVA | 13.709 | 3.506, 53.607 | <0.001 | 8.915 | 1.378, 57.666 | 0.022 |
| SAP |  |  |  |  |  |  |
| Baseline MD | 0.975 | 0.904, 1.051 | 0.505 |  |  |  |
| Baseline VFI | 0.991 | 0.968, 1.014 | 0.449 |  |  |  |
| OCT |  |  |  |  |  |  |
| Baseline average RNFL thickness | 0.982 | 0.932, 1.034 | 0.489 |  |  |  |
| Baseline average macular GCIPL thickness | 0.954 | 0.899, 1.012 | 0.117 |  |  |  |

Factors with P < 0.1 in the univariate analysis were included in the multivariate analysis.

IOP = intraocular pressure; SAP = standard automated perimetry; MD = mean deviation; VFI = visual field index; OCT = optical coherence tomography; RNFL = retinal nerve fiber layer; GCIPL = ganglion cell–inner plexiform layer
